# QuantiFERON-TB Gold *plus* testing for the detection of LTBI among health care workers in major TB hospitals of the Northern Kyrgyz Republic

**DOI:** 10.1186/s12879-022-07149-0

**Published:** 2022-02-23

**Authors:** Caroline Corbett, Gulmira Kalmambetova, Nagira Umetalieva, Sevim Ahmedov, Uladzimir Antonenka, Bakyt Myrzaliev, Evgeni Sahalchyk, Monica Vogel, Abdylat Kadyrov, Harald Hoffmann

**Affiliations:** 1 Institute of Microbiology and Laboratory Medicine, Department IML Red GmbH, WHO, Supranational Tuberculosis Reference Laboratory, Robert-Koch-Allee 2, Gauting, 82131 Munich, Germany; 2 Republican Tuberculosis Reference Laboratory, Bishkek, Kyrgyzstan; 3 USAID, Bureau for Global Health, TB Division, Washington, DC USA; 4 KNCV Branch Office in the Kyrgyz Republic, Bishkek, Kyrgyzstan; 5 Republican Tuberculosis Center, National TB Program, Bishkek, Kyrgyzstan; 6 SYNLAB Gauting, SYNLAB MVZ Humane Genetics, Munich, Germany

**Keywords:** Latent TB infection, LTBI, QuantiFERON, Health care workers

## Abstract

**Background:**

Health care workers (HCW) are at increased risk of TB infection due to their close contact with infected patients with active TB. The objectives of the study were (1) to assess the prevalence of LTBI among HCW in the Northern Kyrgyz Republic, and (2) to determine the association of LTBI with job positions or departments.

**Methods:**

HCWs from four TB hospitals in the Northern Kyrgyz Republic were tested with the interferon-gamma release assay (IGRA) Quantiferon-TB Gold *plus* (QFT) for the detection of an immune response to TB as marker of TB infection. Age was controlled for as a confounder. Univariate and multivariable analysis were performed using logistic regression to assess the association of the risk factors (job position, and department) with having a QTF positive result. Firth’s penalized-likelihood estimates were used to account for the small-sample size. Pairwise comparisons using the Bonferroni correction (conservative) and comparisons without adjusting for multiple comparisons (unadjusted) were used to identify the categories where differences occurred.

**Results:**

QFT yielded valid results for 404 HCW, with 189 (46.7%) having a positive test. In the National Tuberculosis Center there was an increased odds to have a positive QFT test for the position of physician (OR = 8.7, 95%, CI = 1.2–60.5, p = 0.03) and laboratory staff (OR = 19.8, 95% CI = 2.9–135.4, p < 0.01) when administration staff was used as the baseline. When comparing departments for all hospitals combined, laboratories (OR 7.65; 95%CI 2.3–24.9; p < 0.001), smear negative TB (OR 5.90; 95%CI 1.6–21.8; p = 0.008), surgery (OR 3.79; 95%CI 1.3–11.4; p = 0.018), and outpatient clinics (OR 3.80; 95%CI 1.1–13.0; p = 0.03) had higher odds of a positive QFT result than the admin department. Fifteen of the 49 HCW with follow-up tests converted from negative to positive at follow-up testing.

**Conclusions:**

This is the first report on prevalence and risk factors of LTBI for HCW in the Kyrgyz republic, and results indicate there may be an increased risk for LTBI among physicians and laboratory personnel. Further research should investigate gaps of infection control measures particularly for physicians and laboratory staff and lead to further improvement of policies.

## Background

Tuberculosis (TB) affects over 10 million people worldwide and it is estimated that nearly 1.8 billion people are latently infected with TB [1, 2]. Latent TB infection (LTBI) occurs when an individual develops a measurable immune response to TB bacteria, but symptoms and signs of clinical disease have not yet manifested [2]. It is estimated that about 5–15% of individuals with LTBI will progress to active TB at a certain time in their life, and those with active pulmonary TB can transmit infection to up to 15 people [2]. The land-locked Kyrgyz Republic in Central Asia is not only considered a high incidence country (2017 incidence 144/100,000; 7695 TB cases notified), but also has a high burden of drug-resistant (DR)-TB (1372 [43.5%] bacteriologically confirmed MDR/RR-TB among 3,151 bacteriologically confirmed cases in 2017) [3] increasing the importance of effective control measures. Controlling LTBI is considered a key to control the TB epidemic in a country [1, 2]. Latent TB infection can be detected by indirect immunological responses using methods such as the Tuberculin Skin Test (TST), or interferon-gamma release assays (IGRA) such as the QuantiFERON-TB Gold *plus* (QFT; Qiagen, Hilden, Germany). In countries where BCG vaccination (in the Kyrgyz Republic national BCG coverage from 2009 to 2019 was between 96 and 99% [4]) and interference with the TST is common, IGRA have a markedly higher specificity to detect and monitor LTBI [5, 6].

Health care workers (HCW) are at increased risk of TB infection due to their close contact with TB patients, particularly when performing high risk procedures such as cleaning, sample collection, sample testing, intubation, or resuscitation [7, 8]. Although effective training and TB infection control measures should reduce transmission of TB in medical facilities [9], the annual incidence of LTBI among HCW in high prevalence countries is estimated to be as high as 7.2% [10], which is greatly increased over the general public [8, 10, 11]. The high occupational risk to HCW is a problem not only for the individuals themselves, but also to the general public as progression to active TB leads to transmission not only in the community, but also in the hospitals during interactions with patients [11]. In order to develop and implement effective intervention strategies, it is important to determine and understand the actual prevalence and risk of LTBI among HCW in different job positions and departments within hospitals [12]. Additionally, there may be differences between hospitals depending on the effectiveness of institutional infection control measures. Therefore, it is important not only to monitor LTBI infections among HCW, but also to identify areas of increased risk of infection.

Our study was part of the USAID funded *Stop TB Transmission in Hospitals* (StopTTH) project under the Central Asian USAID-Defeat TB initiative. It aimed to (1) assess the prevalence of LTBI among HCW in the major hospitals in the Northern Kyrgyz Republic using QFT, and (2) determine whether specific departments or job positions are associated with increased risk of LTBI.

## Methods

This study was conducted in the four major TB inpatient facilities of the three most Northern regions of the Kyrgyz Republic. Those were the TB hospitals of the National Tuberculosis Center (NTC) in Bishkek, Karabalta, Kemin and Karakol where 351, 104, 45, and 50 staff, respectively, were working in 2018. The NTC is larger than the three other hospitals and constitutes the central TB hospital of the country where advanced and complicated cases can be treated. The other 3 hospitals are rural and of simpler infrastructure.

### Participants

All individuals who worked in the TB hospitals of Karakol, Kemin, Karabalta, and at the National Tuberculosis Center (NTC) in Bishkek were invited to participate in this study during their regular occupational-medical health examination. Participants completed a questionnaire indicating their age, job position, and department in which they worked. All participants had to declare their consent to give blood for IGRA testing, and to use their data including their QFT results for this study. All employees of the hospitals who gave their informed consents were eligible to be enrolled in this study. Employees under 18 years of age were excluded.

### QuantiFERON-TB *Gold plus*

Participants provided 4.0 ml of heparine-blood and all assays were run within three days of collection. As per the manufacturer’s instructions (QuantiFERON-TB *Gold* plus^®^, Qiagen, USA), 1.0 ml blood was distributed among each of the four QFT tubes for negative control (nil), positive control (mitogen), TB1 and TB2 antigens. Results were categorized as positive, negative, or indeterminate as per manufacturer specifications. Blood samples were transported to the respective hospital laboratory in less than three hours before start of incubation (36 ± 1 °C for 16–24 h). Interferon-gamma concentrations were determined by means of the QuantiFERON-TB Gold *plus* ELISA kits using the automated microplate washer ELx50/8 and reader ELx800 (Qiagen-BioTek, Hilden, Germany). A positive result was assigned when TB1 or TB2 minus the corresponding nil-values were ≥ 1.0 IU/ml and > 25% of the nil-value; a borderline positive result was assigned when both TB1 and TB2 minus nil were below 1.0 IU/ml but at least one (TB1 or TB2 minus nil) was above 0.35 IU/ml and > 25% of nil. When mitogen was ≥ 0.5 IU/ml, nil ≤ 8.0 IU/ml, both TB1 and TB2 minus nil < 0.35 IU/ml or < 25% of nil, a negative QFT was assigned. In all other cases the QFT result was called indeterminate.

All participants at the NTC or Karakol were offered up to three follow-up QFT tests performed during the study period (February 2018–June 2019). The first two valid tests taken at least one month apart were used to determine the QFT “status” of the HCW. A positive status (P) was assigned when both tests were positive, a negative status (N) when both tests were negative, a “converter” status (C) when the first test was negative followed by a positive one, and a “reverter” status (R) when the first test was positive followed by a negative one. The stability of respective status was determined when a third QFT test was performed within one month of the second. A stable status was assigned when the third test matches the second test, whereas an instable status was assigned when the third test does not match the second.

All HCW with positive QFT results were examined by experienced phthysiatricians and none of them was diagnosed with active TB.

### Statistical analysis

All statistical analysis was performed using STATA 16.0 (StataCorp LP, College Station, TX). Univariate and multivariable analyses were performed using logistic regression to assess the association of the risk factors (age, job position, and department) with having a QTF positive result. Age was controlled for as a confounder, and separate estimates were made for either job position or department as the risk factor for the outcome of QFT positivity. Administrative staff were used as the baseline as they best represent typical population in the Kyrgyz republic due to limited/minimal contact with TB patients. Due to the “rare event” of TB being detected in the baseline group, Firth’s penalized-likelihood estimates were used for determining the odd’s of QFT positivity to account for the small-sample size bias that is introduced. Pairwise comparisons using the Bonferroni correction (conservative) and comparisons without adjusting for multiple comparisons (unadjusted) were used to identify the categories where differences occur. Confidence intervals were used to determine significance for pairwise comparisons using the Bonferroni correction. Differences between the 4 hospitals were analysed using a mixed effect logistic regression, with site as a random effect, to account for any innate differences that were not measured. Additionally, the NTC was compared against the three other sites combined (Karabalta + Karakol + Kemin) for associations with risk factors; and a students t-test was used to compare the average age between the NTC and the combined other sites. Department differences were only analysed for all four hospitals combined due to decreased sample size, and similarities in department categorization between hospitals.

For follow-up analyses, the second QFT test result was used unless there was less than one month that had elapsed between tests of an individual HCW. In this case, the 3rd test was used if available and more than one month had elapsed. Participants with less than two valid QFT taken at least one month apart were dropped from the follow-up analysis. Therefore, all participants with at least two valid QFT collected at least one month apart were included in the follow-up analysis. Fisher’s exact test and logistic regression were used to assess associations of study sites and age, time between tests, or job position, respectively with conversion or reversion of follow-up tests. Significant associations were those that had a p-value < 0.05 for all analysis.

### Ethical clearance

Permission to conduct the study was obtained from the Ethical Committee of the Kyrgyz Ministry of Health (registration no 01-3/270 from Sep 11, 2017). All participants provided written, informed consent prior to participation.

## Results

### Descriptive results, and controlling for age as a confounder

Of all 550 HCW (73 doctors, 198 nurses, 150 cleaners, 129 others) working in the study hospitals at the time of the study, 409 (74.4%) participants were recruited and had at least one QFT performed (NTC = 222 [63.2%], Karabalta = 100 [96.1%], Kemin = 38 [84.4%], Karakol = 49 [98.0%]). Five tests with indeterminate results (NTC: n = 2; Karabalta: n = 2; Kemin: n = 1) between the ages of 27–63 years were removed from the analysis resulting in 404 HCW that were included. There were five HCW that did not provide age and were not included in the age-controlled analysis. At both the NTC and the other sites combined, nurses had the highest level of recruitment to the study and ranged in age from 21 to 70 years old (Table 1).

**Table 1:** Descriptive characteristics and results of the Firth penalized-likelihood estimates for the effect of health care worker (HCW) position in the study hospitals on being Quantiferon-TB Gold *plus* positive while controlling for age

	NTC									Kara Balta, Karakol, Kemin combined^a^					
Type of HCW	Number of participants n %	Mean age years (range)	QFT Positive n %	OR	95% CI	P-value	KB	KK	KM	Total %	Mean age (range)	QFT Positive n %	OR	95% -CI	P-value
Admin	10 4.5%	37.5 (23–53)	1 10.0%	Base			9	5	1	15 8.2%	48.7 (31–64)	5 33.3%	Base		
Head of department	7 3.2%	56.6 (48–66)	4 57.1%	4.1	0.9–39.4	0.06	2	1	2	5 2.7%	63.0 (57–70)	3 60.0%	2.1	0.3–14.9	0.45
Physician	**23** **10.5%**	**47.3** **(26–82)**	**15** **65.2%**	**8.7**	**1.2–60.5**	0.03*	5	5	2	12 6.5%	43.5 (26–69)	4 33.3%	1.1	0.2–5.2	0.91
Nurse	85 38.6%	42.1 (21–67)	43 50.6%	5.9	1.0–35.4	0.10	26	13	10	49 26.6%	49.0 (29–70)	19 38.8%	1.3	0.4–4.1	0.70
Cleaner	34 15.5%	47.9 (30–64)	20 58.8%	6.01	0.9–39.4	0.06	22	8	10	40 21.7%	49.8 (26–67)	16 40.0%	1.3	0.4–4.2	0.70
Lab	**32** **14.5%**	**38.3** **(22–71)**	**24** **75.0%**	**19.8**	**2.9–135.4**	0.002*	4	6	2	12 6.5%	48.4 (26–66)	5 41.7%	1.4	0.3–6.4	0.66
Technical	17 7.7%	53.1 (38–63)	7 41.2%	2.6	0.3–19.1	0.356	30	9	10	49 26.6%	48.4 (26–68)	17 34.7%	1.1	0.3–3.4	0.96
Other	12 5.5%	54.6 (42–66)	5 41.7%	2.0	0.2–17.1	0.52	0	2	0	2 1.1%	35 (NA)	1 50.0%	NA	NA	NA
Total	220 100.0%	44.7 (21–82)	119 54.1%				98	49	37	184 100.0%	48.9 (25–70)	70 38.0%			

*NTC* National Tuberculosis Centre, *QFT* Quantiferon-TB Gold *plus*, *KB* Karabalta, *KK* Karakol, *KM* Kemin

^a^Numbers of participants from Kara Balta, Karakol and Kemin are given as reference only, as total number of HCW in each position were combined among these three sites for this analysis

*Significant with p < 0.05

The average age of HCW at the NTC (44.7 years) was lower than the other sites combined (48.9 years; p < 0.001). Additionally, there was no random effect of site on the outcome of the QTF test (p = 0.063) based on the mixed effects logistic regression including age and job position as dependent variables for QFT when analysing all four sites independently. However, there were differences in the mixed effects logistic regression when comparing the NTC and the three other sites combined (p = 0.02); therefore, logistical analyses were split into two separate analysis by site (Table 1).

In univariate analysis, increasing age per year was associated with a positive quantiferon result (OR = 1.02 per year, 95% CI = 1.00–1.03, p-value < 0.001), and this effect occurred among all job positions. Age was controlled for in the analysis due to the association between job position (p < 0.001) and QFT positivity.

### Job position as a risk factor

There were 189 (46.7%) HCW that had a positive QTF result in total at all sites. In the NTC, physicians (OR = 8.7, 95%, CI = 1.2–60.5, p = 0.03) and laboratory staff (OR = 19.8, 95% CI = 2.9–135.4, p < 0.01) had increased odds to have positive QFTs than administrative staff. Nurses (OR = 5.9, 95% CI = 1.0–35.4, p = 0.05) and cleaners (OR = 6.01, 95% CI = 0.9–39.4, p = 0.06) showed trends towards increased odds of positivity (Table 1). In the conservative pairwise comparisons at the NTC for all job positions with the Bonferroni correction, no position was any different than another for testing positive. There was no effect of job position on the QTF results for the combined group of Karabalta, Karakol and Kemin (Table 1).

### Department as a risk factor

In order to compensate for small sample size, all hospitals were combined for comparisons of departments. The laboratories (OR 7.65; 95%CI 2.3–24.9; p < 0.001), smear negative TB (OR 5.90; 95%CI 1.6–21.8; p = 0.008), surgery (OR 3.79; 95%CI 1.3–11.4; p = 0.018), and outpatient clinics (OR 3.80; 95%CI 1.1–13.0; p = 0.03) had higher odds of a positive QFT result than the admin department (Table 2). With a conservative pairwise comparison adjusting for multiple comparisons, only the laboratory compared to administrative staff was significantly different (contrast = 2.03, Bonferroni 95% CI = 0.04–4.02).

**Table 2:** Logistic regression results for differences between departments for the National Tuberculosis Centre (NTC), Karabalta, Karakol and Kemin combined, when controlling for age

Department	Number	QFT positive		OR	95% Confidence interval	P-value
		n	%			
Admin/monitoring	23	5	21.7%	Base		
Surgery	55	28	50.9%	3.79	1.3–11.4	0.018*
Lab	39	26	66.7%	7.65	2.3–24.9	0.001*
Drug resistant TB	98	46	46.9%	2.82	0.995–8.0	0.051^**+**^
Outpatient clinic	26	14	53.8%	3.80	1.1–13.0	0.033*
Pediatrics	13	4	30.8%	1.32	0.3–5.8	0.71
Intensive care unit	11	5	45.5%	2.72	0.6–12.1	0.19
Smear positive TB	16	8	50.0%	2.92	0.7–11.4	0.12
Smear negative TB	21	13	61.9%	5.90	1.6–21.8	0.008*
General TB	82	31	37.8%	1.77	0.6–5.1	0.29
Engineering and technical staff	15	6	40.0%	2.03	0.5–8.1	0.32
Total	399					

^**+**^Trend; *statistical significance

### Follow-up

There were 52 HCW that had at least one additional QFT. Three HCW were removed from analysis as follow-up tests were not at least one month following first QFT, resulting in 49 HCW included in the follow-up analysis (NTC = 28, Karakol = 21; Table 3). Follow-up tests were taken on average 160 days after the first test (range 69–257 days). Seventeen HCW had a positive QFT at both times (P status), 12 had both negative QFT (N status), 15 converted from negative to positive (C status), and five reverted from positive to negative (R status). Site (p = 0.27), age (p = 0.47), time between tests (p = 0.82), or job position (p = 0.75) had no effects on the follow-up status. There was no association with having a borderline positive first test and the outcome of the 2nd test being either positive, or negative (p = 0.16). Of the 30 participants that had a 3rd valid QFT performed, 9 of 10 (90%) and all (13/13, 100%) participants of the C and P status, respectively, remained positive. Of those with N status (6/6, 100%) remained negative for the 3rd QFT (Table 3).

**Table 3:** Follow-up QuantiFERON-TB Gold *plus* tests of HCW indicating stable and instable test results for HCW

Status^a^	1st ≥ 2nd QFT	HCW with status	HCW with 3rd QFT performed	3rd QFT results	
				Positive n (%)	Negative n (%)
P	Positive ≥ positive	17	13	13^b^ (100%)	0
N	Negative ≥ negative	12	6	0	6^b^ (100%)
C	Negative ≥ positive	15	10	9^b^ (90%)	1
R	Positive ≥ negative	5	1	1	0
Total		49	30	23	7

Stable status was assigned when the 3rd test matched the 2nd, and instable status when it did not

^a^Positive (P); Negative (N); Converter (C); Reverter (R)

^b^Stable status based on 3rd QFT test

## Discussion

Our study is the first of its kind to describe prevalence and risk factors of LTBI among HCW in Central Asia. It supports the commonly reported findings that job position in the hospital has an effect on QTF positivity, and there is a higher prevalence of LTBI among HCW than the general public [11, 13, 14]. The prevalence of 46.7% QFT positive HCW in the current study was markedly higher than the LTBI prevalence of 3.9% reported from low-incidence countries [10] and 12–28% reported from the medium-incidence country Iraq [7], but well within the range of the 34–60% LTBI among HCW reported from other high-incidence countries [11]. It should be considered that the cited studies investigated HCW in multi-disciplinary hospitals, while the participants of the current study were exclusively working with TB patients.

WHO guidelines for infection control among HCW were released in 2005, which have since been updated in 2019 [15], and have also been implemented in most health care institutions in the Kyrgyz Republic which should have had a meaningful impact on transmission and prevalence of LTBI [9, 16]. However, the current study indicates HCW may have an increased risk of LTBI, especially doctors, laboratory personnel, and those working in the drug resistance and smear-negative wards in the Kyrgyz Republic; although these differences were no longer significant when adjusting for multiple comparisons in pairwise analysis (excluding personnel in the department “laboratory”). Research conducted in Brazil, South Africa, and similar high TB burden countries have found that there is not a lack of protocols or equipment leading to gaps in infection control, but rather a lack of understanding of and adherence to infection control standards, uptake of prevention training, and efficient monitoring [17–19]. HCW of the DR-TB and smear negative pulmonary TB departments had significantly higher odds of being QFT positive, although infection control measures should be particularly strict in the DR-TB ward, and risk of infection relatively low in the department of smear negative TB. Our findings suggest that work safety conditions might not yet be optimal in Kyrgyz TB health facilities, but also that HCW are not yet fully compliantly following the infection control standards especially where the perceived risk of transmission is presumed to be comparably low.

Of the 49 HCW that provided valid follow-up QFT during the 17-month study period, 15 (30.6%) converted from negative to positive which may reflect the high risk of infection among these professions. These results correspond to findings in previous studies whereby 11% of HCW in a medical institute in a rural area of Central India (incidence rate of over 300/100,000) converted with QFT over a period of 1 year [20] and 10% of HCW converted in Malaysia with an incidence rate around 63 per 100,000 population [21]. Although serial testing with IGRA can be influenced by various factors [20, 22], QFT has repeatedly demonstrated its superior reliability over TST in systematically BCG vaccinated populations with high incidence of TB [23]. Although there was no association with age, site, time between tests, or job position, the majority of patients that converted to positive remained positive at follow-up testing, strongly indicating new LTBI infections which may progress to active TB. In a setting with one third of TB patients having multi-drug resistant forms of the disease, active TB among HCW and their potential to spread the infection to vulnerable patient groups is of particular public health concern.

There were five individuals with tests that reverted from positive to negative at follow-up, and it has been suggested that borderline results (falling within an “uncertain range” of e.g., 0.22–0.71 IU/ml) may be more likely to change at follow-up [20, 24]; however, we did not see an association with borderline positivity of the first and the outcome of the second test. The apparent randomness and inconsistency of reversions at follow-up has been identified in previous research [25, 26] and changes have not been explained by occupational exposure in HCW [27]. Therefore, reversions identified in serial QTF testing should be interpreted with caution.

The findings of this study are of particular impact for public health and TB control projects in Central Asia as they provide the first insight in occupation specific prevalence of LTBI; however, certain limitations of the study should be kept in mind when interpreting the results. Firstly, there is a lack of information regarding the duration that the HCWs have been working in health, particularly the TB sector. It has been documented in previous research that there is an increased risk of positivity as length of employment in pulmonary hospitals increases [14]. This risk factor should be considered in future studies investigating TB transmission to HCW in Central Asia and the effectiveness of implemented control measures. There is also a lack of information regarding potential TB contacts outside the hospitals as the collection of such data was not covered by the occupational health screening program of this study. The risk of not work-related TB infection would however have been equal for all participants while we observed a clearly increased risk of LTBI only for those with regular contacts to TB patients. It should be considered that the follow-up test interval of at least one month in the current study is much shorter than most studies where investigations for serial QTF testing were conducted at intervals of three [24] to 18 months [20]. However, this effect on the results should be minimal as time between tests was not found to predict conversion status. There is likely a high amount of variability among HCW at the NTC, as the wide confidence intervals are not reflected in the smaller sample size of the three other hospitals combined. Additionally, apparent differences between job positions at the NTC for risk of QFT are no longer significant when adjusting for multiple comparison, and further research is required to fully understand HCW risk. Due to its relatively small sample size, 95% confidence intervals were large for some groups of HCW weakening statements regarding the risk of LTBI although ORs might be well above 4.0 and trends might be observable. Larger sample sizes should be considered in future studies when investigating health care facilities in Central Asia. As the baseline positivity rates of urban and rural populations are unknown for the Kyrgyz Republic, we had to use administrative staff as base group which might however bring in some bias. Our study has focussed on TB hospitals and laboratories only; population based studies would help estimate the real LTBI risk of HCW compared to the normal population in similar environment. Studies in other types of hospitals and health facilities would increase the representativeness of the results for all Kyrgyz HCWs. Simultaneous investigations of implemented and applied infection control measures would allow to measure the effectiveness and protective value of individual or bundles of measures.

## Conclusions

This is the first international report on prevalence of LTBI among HCWs in the Kyrgyz Republic established by means of modern IGRA, and results indicate an increased risk of TB infection for TB physicians, laboratory staff, and those working in the drug resistant and smear-negative TB departments. Although all Kyrgyz hospitals have implemented infection control measures, our findings suggest that the infection control policies may be insufficient, or HCW may not be consequently adhering to the recommended standards. This research can be used to guide targeted interventions with higher efficiency, ultimately leading to a decrease in the risk of TB for HCWs and to interrupt TB infection cycles in all Central Asian Hospitals. Consideration should be made to include IGRA testing in screening algorithms among HCWs in order to monitor the success of intensified infection control efforts.

## Data Availability

The datasets used and/or analysed during the current study are available from the corresponding author on reasonable request.

## Abbreviations

C: Converter status
CI: Confidence interval
DR-TB: Drug resistant tuberculosis
HCW: Health care worker
IGRA: Interferon-gamma release assay
LTBI: Latent tuberculosis infection
N: Negative status
NTC: National Tuberculosis Center
OR: Odds ratio
P: Positive status
QFT: Quantiferon-TB Gold *plus* test
R: Reverter status
TB: Tuberculosis
TST: Tuberculin skin test
